# Effect of Mechanical Stirring on High-Speed GMAW Hump Bead

**DOI:** 10.3390/ma16124493

**Published:** 2023-06-20

**Authors:** Jun Xiao, Xiaolei Wang, Shengnan Gai, Shujun Chen, Wenhao Huang

**Affiliations:** 1 Engineering Research Center of Advanced Manufacturing Technology for Automotive Components, Ministry of Education, Beijing University of Technology, Beijing 100124, China; jun.xiao@bjut.edu.cn (J.X.);; 2 Welding Equipment R&D Center, Beijing University of Technology, Beijing 100124, China; 3 College of Mechanical and Electrical Engineering, Wenzhou University, Wenzhou 325035, China; huangwenhaojay@163.com

**Keywords:** high-speed GMAW, mechanical stirring, humping bead, weld pool flow

## Abstract

High-speed GMAW tends to be accompanied by periodic humping defects, thereby reducing the weld bead quality. A new method was proposed to actively control the weld pool flow for eliminating humping defects. A high-melting point solid pin was designed and inserted into the weld pool to stir the liquid metal during the welding process. The characteristics of the backward molten metal flow were extracted and compared by a high-speed camera. Combined with particle tracing technology, the momentum of the backward metal flow was calculated and analyzed, and the mechanism of hump suppression in high speed GMAW was further revealed. The stirring pin interacted with the liquid molten pool, resulting in a vortex zone behind the stirring pin, which significantly reduced the momentum of the backward molten metal flow, and thus it inhibited the formation of humping beads.

## 1. Introduction

As one of the most important arc welding processes in the manufacturing industry, gas metal arc welding (GMAW) has the advantages of high automation, adaptability, and low production cost. Hump welding is one of the commonly formed defects in high-speed GMAW, and it has become a bottleneck to improve production efficiency [1,2]. Therefore, understanding the formation process and mechanism of the hump can help us to take action to prevent its occurrence. At present, it is widely recognized that the reverse metal flow in the molten pool at a high speed is the main cause of hump formation. Nguyen et al. [3] confirmed that the powerful momentum of the reverse flow of molten metal in the molten pool was the main factor causing the hump by collecting sequential images during the hump formation. With the maturity of hump research, many scholars simulated hump welding by numerical calculations [4,5,6,7,8], calculated the velocity distribution of liquid metal in the molten pool, observed the flow state of molten pool more intuitively, and verified the effect of reverse metal flow on hump formation. If the characteristics of the reverse metal flow are directly extracted by experimental method, the relationship between the liquid metal flow and welding parameters and forming can be quantitatively analyzed.

Zähr et al. [9] studied the influence of protective gas composition on humping in TIG welding and found that the addition of helium increased the heat transfer of the workpiece, thus increasing the welding speed without the hump. Based on the current research on the formation of the hump bead, many improved arc welding processes or equipment have been reported to prevent the hump bead. For example, double- or three-wire GMAWs [10,11,12,13] are considered because the arc pressure and the impact force of the droplet were evenly distributed, and the relative value was small in the double- or three-wire co-melting cell, the interconnected molten pool act as a coordinated buffer, which can delay the reflux tendency of liquid metal and inhibit hump defect. Double electrode GMAW [14], due to bypass diversion, resulting in part of the main current being flown to the bypass arc, which was applied to heat and melt the bypass wire, which improves the welding rate and avoids the hump. TIG-MIG hybrid welding [15,16] and Laser GMAW hybrid welding [17,18] can improve the fluid flow and the heat transfer in the molten pool, thereby reducing the momentum of backward molten metal flow and inhibiting the hump. However, the double electrodes or wires and hybrid heat source welding not only increase equipment investment, but they also make equipment more complex and difficult to operate.

The Shandong University team applied an external transverse magnetic field (TMF) to the molten pool, using electromagnetic forces generated by electromagnetic induction to suppress the flow of reverse liquid metal and successfully prevented the hump beads [19,20]. Then, combining the visual sensing system with the tracing particles, a two-dimensional fluid flow field [21,22,23] was measured on the surface of the molten pool. The relationship between the reverse liquid metal and the hump was quantitatively analyzed. The additional electromagnetic force produced by the interaction of the external magnetic field and the welding current can inhibit the flow of reverse metal in the weld pool. Studies have shown that the maximum velocity of the tracing particles dropped below the threshold level when a magnetic field was applied. However, the way in which magnetic fields are added is still relatively cumbersome and requires some cost.

Wang et al. [24] explored the effect of compensating gas on hump defects in TW-GIA and revealed its effect on molten pool flow and arc stability. The results have shown that the compensating gas with an injection angle of 90° has the ability to suppress the hump bead and reduces the oxygen content of the weld.

In this study, a new mechanical stirring process was proposed for the first time. Tungsten rods are designed to be desired shapes. The designed tungsten rod was inserted directly into the molten pool and rotated by the stepping motor to stir with the welding. Its equipment is simple, and its effect on the molten pool has great advantages in practical application. At the same time, the velocity of reverse molten metal flow was obtained by using tracer technique. By calculating the velocity of reverse metal without stirring pin and mechanical stirring, respectively, the influence of mechanical stirring on the flow of metal liquid in a molten pool was quantitatively analyzed, and the new mechanism of hump suppression was further revealed.

## 2. Experimental System

### 2.1. Mechanical Stirring System

**Figure 1:**
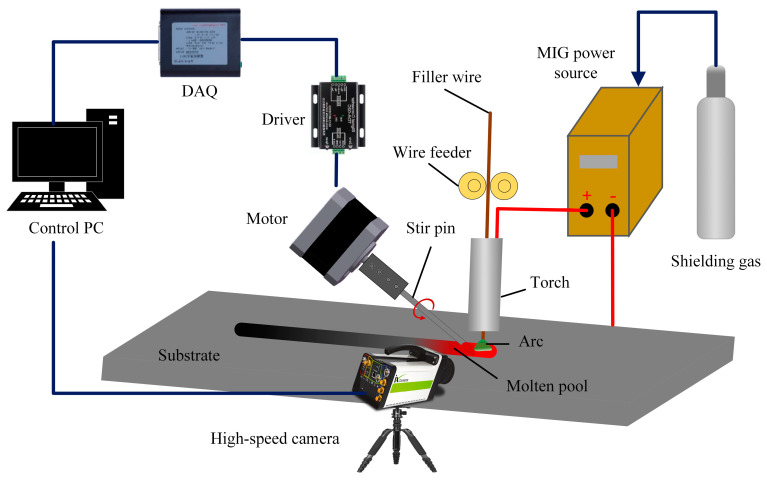
Schematic diagram of the experimental system.

In order to study the hump bead, a high-speed GMAW stirring system was established, as shown in Figure 1, which consists of stirring equipment, MIG power source, a data acquisition system, and a computer control system. The high-speed camera is controlled by the computer to complete data acquisition, the step motor was joined to the tungsten pin through the joining mechanism, and the tungsten pin was rotated to realize the process of stirring with welding.

In this study, bead-on-plate flat welding experiments were carried out on a low carbon steel (Q235) base metal with a dimension of 200 mm × 60 mm × 3 mm. Argon (99.999% purity) was also used as a protective gas for the arc and welds with a flow rate of 20 L/min. The wire used is an ER50-6 carbon steel wire with a diameter of 1.2 mm, and the composition of the wire and the mechanical properties of the base metal are shown in Table 1 and Table 2. It is noteworthy that the rotation of the stirring pin affects the arc plasma, making it unstable, thereby affecting the weld formation. Therefore, after many experiments, the distance between the stir pin and the center of the wire was fixed to be 4 mm. Due to the high hardness of the tungsten pin and its poor machinability, this study only focuses on the simple shape of the cone-shaped stirring pin. The cone-shaped stirring pin can continuously introduce force into the liquid molten pool and can change the flow state in the molten pool, which will hopefully reduce the flow velocity of the reverse liquid metal and achieve the effect of hump suppression. Table 3 lists other process parameters used in the experiment.

### 2.2. Observe the Convective Patterns on the Weld Pool Surfaces

**Figure 2:**
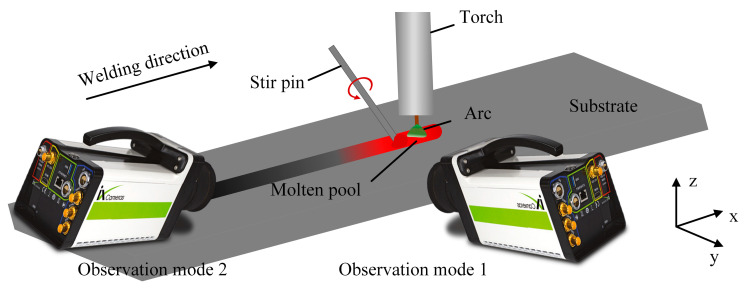
Schematic illustration of the experimental setup to observe the convective patterns on weld pool surfaces.

The influence of fluid flow velocity on the hump was quantitatively studied by observing the motion of tracing particles. A high-speed camera (iX-Cameras, i-Speed 720) was connected to the computer, and photo shooting was performed at a speed of 5000 fps. A narrow band pass filter of 630 ± 20 nm was installed in front of the lens to reduce intense arc interference. Zirconia (ZrO_2_) has a much lower density (5.85 g/cm^3^) than iron solution and does not react with iron. In addition, the melting point of zirconia (2700 °C) is higher than that of iron and becomes an ideal tracer particle. In order to accurately reflect the flow velocity of surface tracing particles, 0.3 mm zirconia particles were used as tracers. Before testing, a blind hole was drilled with a diameter of 1.2 mm and a depth of 1mm every 15 mm along the centerline of the top of the workpiece, and then the particles were placed into the blind hole. The upper part of the blind hole was sealed with a small piece of wire to prevent the tracer particle from flying out under the force of the protective gas and the arc. Figure 2 is a schematic illustration of the experimental setup to observe the convective patterns on the surface of the molten pool. In order to fully observe the motion of tracing particles, the image of the molten pool was taken from two angles: welding direction and vertical welding direction, respectively. By extracting the coordinates of the tracing particles in the image, the trajectory of the tracer particle was obtained, and its flow velocity was calculated.

## 3. Results and Discussions

### 3.1. Observation of Weld Pool Behavior

**Figure 3:**
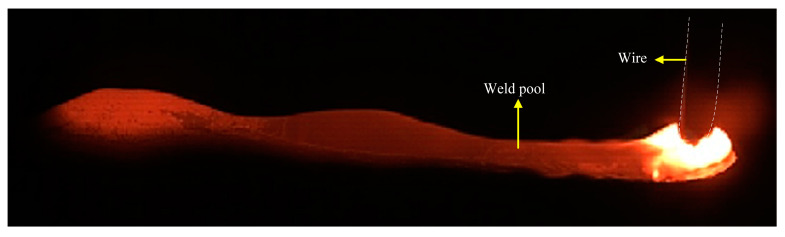
Traditional high-speed GMAW molten pool image.

**Figure 4:**
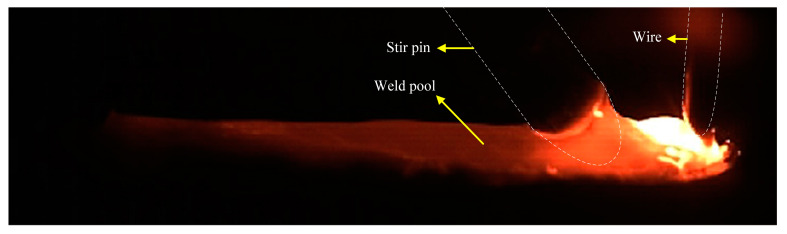
Image of high-speed GMAW molten pool under mechanical stirring.

This study adopts a new method of mechanical stirring. By changing the rotation speed of the stirring pin, an additional force source was applied to the molten pool to change the flow behavior and temperature field of the liquid metal in the molten pool. The flow pattern of the molten pool was compared with that of the molten pool with or without mechanical stirring. Figure 3 shows an image of a conventional high-speed GMAW molten pool, showing obvious hump defects. The results show that the formation of the hump bead passed through three stages: initial stage, growth stage, and solidification stage. In the initial stage, under the combined effect of arc force and droplet impact, the front surface of the molten pool was depressed, creating a high momentum reverse metal flow toward the end of the molten pool. The dynamic changes in the arc force and the impact force of the droplet cause the liquid metal to flow towards the molten pool tail wave undulation. In the growth stage, the hump continued to grow as the metal was continuously filled and the molten parent material was filled. During the solidification stage, as the heat source moved forwardly, the molten pool became elongated, and the insufficient heat was available at the end of the molten pool to solidify first, preventing the liquid metal from continuing to flow behind the pool to form a crest. Finally, the liquid metal at the crest all solidifies to form a hump. Figure 4 shows the image of a high-speed GMAW molten pool under mechanical stirring. It can be seen from the images taken by a high-speed camera that the molten pool tends to be flat, and the hump disappears after mechanical stirring. Contrast was performed in relation to the stirred or unstirred images to show the reduction in the size of the molten pool after stirring. The results show that mechanical stirring can inhibit the hump. At the same time, stirring can promote molten pool heat dissipation, reduce the weld pool temperature, and reduce the weld pool size.

**Figure 5:**
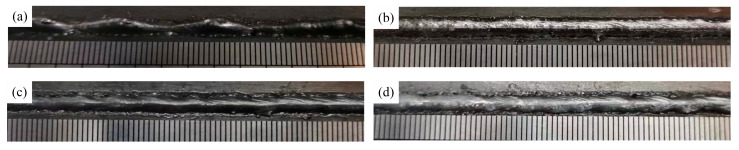
Effect of mechanical stirring on weld forming. (**a**), without stir pin (**b**), 0 r/min (**c**), 500 r/min (**d**), 1000 r/min.

Figure 5 shows the effect of mechanical stirring on bead forming at a welding speed of 2.25 m/min. It can be seen that a typical hump weld bead is formed without stirring pin, as shown in Figure 5a. When the pin keeps static (i.e., 0 r/min), the weld bead becomes uniform and continuous, and the hump is suppressed due to the blocking effect of the pin, which inhibits the backward flow of liquid metal, as shown in Figure 5b. Influenced by the machining accuracy of the pin connector, resulting in eccentric rotation of the pin, the stirring process is unstable, and the weld bead forming is not ideal when the molten pool is stirred with different pin rotation speed, but it still has an inhibitory effect on the hump, as shown in Figure 5c,d.

### 3.2. Influence of Mechanical Stirring on Molten Pool Flow

**Figure 6:**
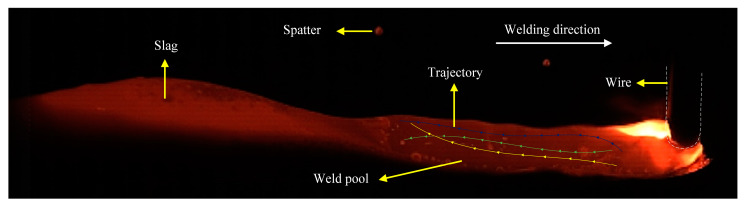
Flow pattern of the molten pool surface without stirring.

**Figure 7:**
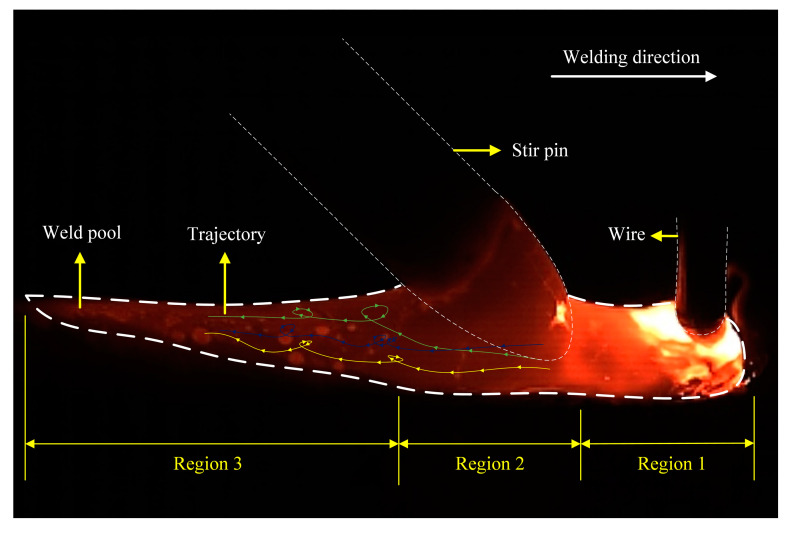
Flow pattern of the surface of the molten pool under mechanical stirring.

At present, many scholars believe that high momentum reverse liquid metal is the main mechanism of hump formation. Therefore, all methods capable of inhibiting the flow velocity of reverse liquid metals can inhibit the occurrence of the hump. In order to clarify the effect of mechanical stirring on reverse liquid metal, in this section, the flow pattern of liquid metal on the surface of the molten pool is reflected by tracking the flow trajectory of tracing particles. Figure 6 shows the flow pattern on the surface of the molten pool without a stirring pin. The zirconia particles flowed backward by the arc force and the droplet impact force and were eventually blocked by the slag behind the molten pool. Figure 7 shows the flow pattern of the surface of the molten pool under mechanical stirring. The molten pool is divided into three parts: the arc influence zone, corresponding to region 1, the stirring pin influence zone, corresponding to region 2, and the liquid molten pool, corresponding to region 3 behind the stirring pin. By tracing zirconia particle motion, it can be seen that the zirconia particles rotate and move under the effect of mechanical stirring, and a vortex zone is formed behind the stirring pin. Therefore, the rotation of the stirring pin changes the flow state in the molten pool, decreasing the reverse liquid metal flow velocity and eventually suppressing the hump.

**Figure 8:**
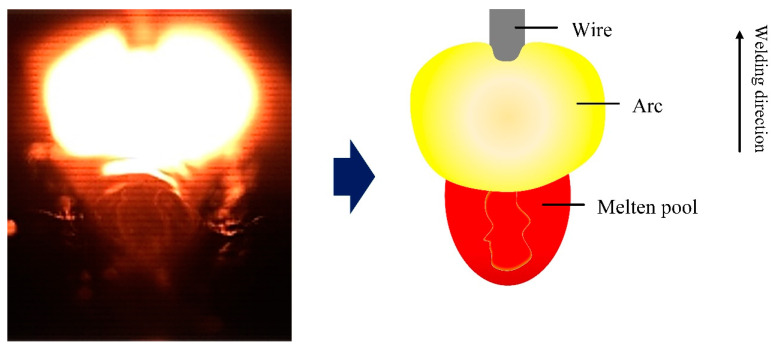
Flow pattern of welding molten pool surface in welding direction without stirring.

**Figure 9:**
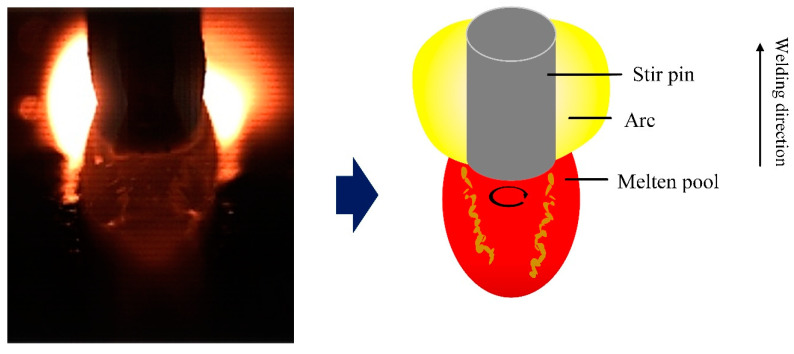
Flow pattern of welding molten pool surface in the direction of welding under mechanical stirring.

The viewing angle of the surface flow pattern of the molten pool was observed in the direction of vertical welding. Only part of the liquid molten pool can be seen. It does not accurately reflect the real flow of the molten pool. Therefore, this study combines with the viewing angle along the welding direction to observe the flow state of the entire liquid molten pool surface. By tracking the movement of zirconia particles on the surface of the molten pool, the complete flow pattern of the surface of the molten pool can be described. Figure 8 shows that the flow pattern of the surface of the molten pool was observed from the angle of view along the welding direction without stirring. Based on the addition of the filter, the arc light was filtered out, sadly, covering part of the front of the molten pool and not being able to be observed. The image of the molten pool visualized from the rear shows that the zirconia particles flowed backward rapidly without stirring, while the high momentum reverse metal in the molten pool flowed backward at a speed higher than the molten pool surface. Figure 9 shows the flow pattern of the viewing angle molten pool surface in the welding direction under mechanical stirring. The strong arc light was blocked by the stirring pin, and the image of the molten pool behind the stirring pin was clearly visible. It can be seen from the image of the molten pool that the reverse liquid metal with high momentum is divided into two by the stirring pin. Simultaneously, by observing the movement of zirconia particles, the zirconia particles were rotated after stirring pin. The above phenomenon shows that a whirlpool was produced on the back side of the stirring pin, which prevents the flow of zirconia particles on the surface of the molten pool. Therefore, it is proved, once again, that under mechanical stirring, the vortex zone was formed behind the stirring pin, which can inhibit the flow of reverse liquid metal.

**Figure 10:**
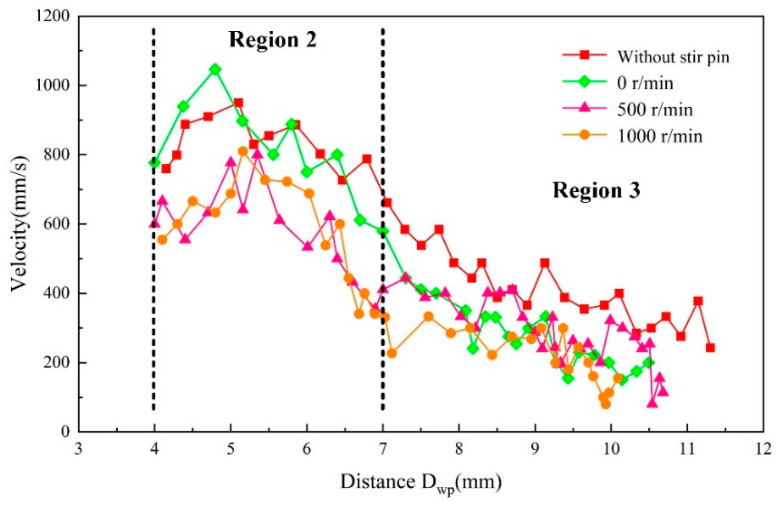
Effect of mechanical stirring on the flow velocity of tracing particles.

In order to further reveal the new mechanism of hump suppression by mechanical stirring of high-speed GMAW, the momentum of metal reverse flow on the surface of molten pool was calculated and analyzed by extracting the pixel coordinates of tracing particles from high-speed camera images. The distance was customized between the weld center and the tracer particle as *D_wp_*. Zirconia particles cannot be observed within the *D_wp_* = 0–4 mm due to strong arc interference. Therefore, we count the flow velocity of zirconia particles after *D_wp_* = 4 mm. Figure 10 shows the effect of mechanical stirring on the flow velocity of tracing particles, where velocity is the absolute value (magnitude). The results show that the flow velocity of zirconia particles gradually increased to 950 mm/s within *D_wp_* = 0–5 mm by the arc force and the droplet impact force under traditional high speed GMAW. Then, the speed gradually decreased. Under mechanical stirring, when the stirring pin did not turn (0 r/min), the flow velocity of zirconia particles within the *D_wp_* = 0–5 mm exceeded the velocity peak of traditional high-speed GMAW and increased to 1046 mm/s. Within the *D_wp_* = 5–7 mm, the flow velocity of zirconia particles decreased gradually. After *D_wp_* = 7 mm, the vortex within the vortex zone again reduced the flow velocity of zirconia particles. In contrast to the case where the stirring pin does not rotate, in the impact area of the stir pin (*D_wp_* = 4–7 mm), the rotation of the stirring pin significantly reduced the flow rate of zirconia particles, indicating that stir pin rotation can reduce the flow velocity of the reverse liquid metal. Comparing the stirring pin rotation, it could be seen that the flow velocity of zirconia particles increased to 800 mm/s and 810 mm/s when the stirring pin speed was 500 r/min and 1000 r/min, respectively, and the velocity change pattern of zirconia particles was similar for both. There was a rotation speed threshold for the stirring action of the stirring pin in the molten pool, and the stirring intensity stopped increasing after this value.

**Figure 11:**
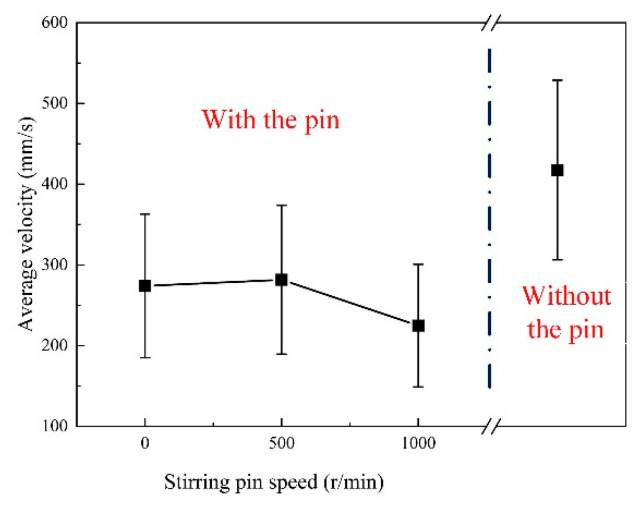
Comparison of average velocity with or without stirring pin.

By calculating the average velocity of region 3 (*D_wp_* = 7–12 mm), as shown in Figure 11, the flow velocity of zirconia particles under mechanical stirring was significantly lower compared to that without stirring pin, indicating that the average velocity of zirconia particles in region 3 decreased with the increase in stirring pin speed. Therefore, the vortex zone produced by mechanical stirring can reduce the flow velocity of the reverse liquid metal, and as the stirring pin speed increases, the more effective the vortex zone is in reducing the flow velocity.

### 3.3. Suppression Mechanism of Humping Bead

**Figure 12:**
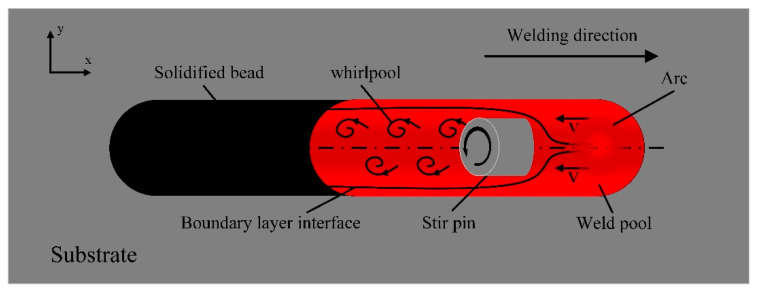
Schematic diagram of cylindrical flow under mechanical stirring.

The partial stirring pin inserted into the molten pool at 45 degrees was reduced to a cylinder and studied as a “cylindrical flow” problem, as shown in Figure 12. The surface flow mode of directly mechanically stirred molten pool can be divided into the following four stages: in the first stage, under the combined action of the arc force and the impact force of the droplet, the surface of the molten pool in front of the pool is depressed, forming high momentum reverse metal flow moving towards the tail of the molten pool. In the second stage, as the welding proceeded, the reverse liquid metal was shunted due to the blocking of the stirring pin, and part of the kinetic energy is lost. When the reverse liquid metal surrounds the front half of the cylinder, part of the pressure was converted into kinetic energy, increasing the velocity of the tracer particle. When the reverse liquid metal surrounds the back half of the cylinder, part of the kinetic energy was converted into pressure energy, part of the kinetic energy was converted into the potential energy of the tracing particles, and the velocity of the tracer particle was reduced. In the third stage, when the reverse liquid metal flows to the back of the cylinder, the boundary layer was separated from the surface of the cylinder, causing the vortex zone to form behind the cylinder. Tracer particle velocity manifests as a decrease. In the fourth phase, the flow velocity of tracing particles continued to decrease and eventually solidify. Under the combined action of the blocking of the stir pin and the eddy flow zone formed after the stir pin, the flow velocity of the reverse liquid metal was reduced, and finally the hump was suppressed.

## 4. Conclusions

This paper mainly studied the suppression effect of mechanical stirring on hump defects at different stirring pin speeds, and conclusions are drawn as follows.

1. A new method that is to use a stirring tungsten pin inserted directly into the molten pool is proposed for achieving better bead formation in the high-speed GMAW process. The results show that the mechanical stirring of the weld pool could successfully suppress the occurrence of the hump defect.
2. The flow velocity of reverse metal flow of the weld pool was analyzed by measuring the motion of the tracing particles. Under the direct mechanical stirring, the flow pattern in the molten pool was changed, and the flow velocity of the backward metal flow was significantly weakened. That is the key mechanism of suppressing the hump defect.
3. The existence of vortex zone behind the stirring pin was proved by tracking the trajectory of tracing particles and the rotating motion of tracing particles in molten pool under mechanical stirring. The vortex zone contributes to weakening the backward metal flow.

## Figures and Tables

**Table 1:** ER50-6 welding wire chemical composition (wt %).

	Fe	C	Mn	Si	P	S	Cr	Ni	Cu	Mo	Ti
ER50-6	Bal.	0.08	1.34	0.14	0.015	0.013	0.021	0.011	0.14	0.38	0.06

**Table 2:** Q235 mechanical properties.

	Tensile Strength (MPa)	Yield Point (MPa)	Elongation (%)
Q235	375–500	≥235	≥25

**Table 3:** Welding parameters.

Parameters	Values	Units
Arc current *I*	220	A
Arc voltage *U*	21	V
Welding speed *v*	2.25	m/min
Stirring speed *n*	0–1000	r/min
Stir pin diameter *d*	3	mm
Stirring pin angle *θ*	45	°

## Data Availability

Data not available due to privacy or ethical restrictions.
